# Atomic layer deposition, a unique method for the preparation of energy conversion devices

**DOI:** 10.3762/bjnano.5.26

**Published:** 2014-03-05

**Authors:** Julien Bachmann

**Affiliations:** 1 Institute of Inorganic Chemistry, Friedrich-Alexander University of Erlangen-Nürnberg, Egerlandstrasse 1, 91058 Erlangen, Germany

**Keywords:** atomic layer deposition, batteries, energy conversion, electrochemistry, electrolysis, fuel cells, photovoltaics, solar cells, thin films

Most of the technical development of the 19th and 20th centuries relied on thermal engines to generate mechanical or electrical work from the combustion of fossil fuels [1]. This strategy of energy production is not renewable, in that finite resources are consumed and greenhouse gases are emitted, and it is also fundamentally inefficient as defined by Carnot. In a more modern strategy which circumvents those disadvantages of thermal machines, energy is converted directly from solar (or some other renewable source) to its electrical or chemical form [2]. Here, fuels still play a fundamental role as energy carriers for the storage and the regulation of the electrical power grid, but they are converted to other energy forms by electrochemical methods rather than thermal engines.

**Figure 1:**
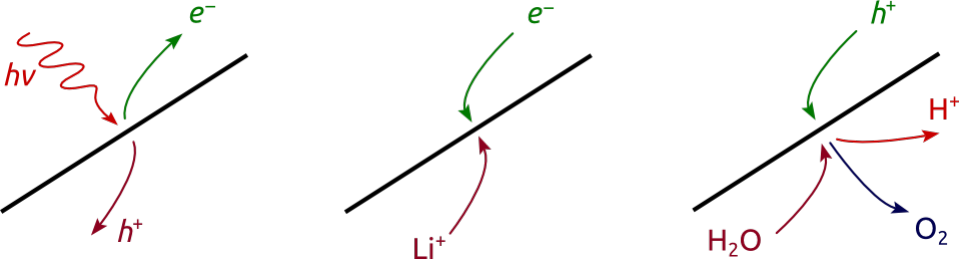
Nature of the charge carriers combining or separating at an interface in a solar cell (left), a lithium ion battery (center), and the water oxidation electrode of an electrolyzer (or the oxygen-evolving complex in photosynthesis, right).

The interconversion of energy between light and electrical forms (in solar cells and light-emitting diodes), between light and chemical forms (photosynthesis and chemiluminescence), and between chemical and electrical forms (batteries, electrolyzers, fuel cells, respiration) always relies on the transport of charge carriers towards an interface and away from it, combined with the transfer of electrons at the interface. This electron transfer, the most fundamental energy-converting single event, occurs at the interface between two phases, which can have various identities depending on the type of device. In most solar cells these two phases are two solid semiconductors, in batteries and fuel cells they are usually a solid and an electrolytic liquid, and in the ‘light reactions’ of photosynthesis the two phases consist of two liquids separated by a lipidic membrane. The nature of the charge carriers that transport electrons between the bulk and the interface varies accordingly: electrons and holes in semiconductors, molecules and ions in electrolytes. Figure 1 summarizes the particular types of charge and energy carriers in a solar cell (left), an electrode of a lithium ion battery (center), and the water oxidation electrode of an electrolyzer (right).

**Figure 2:**
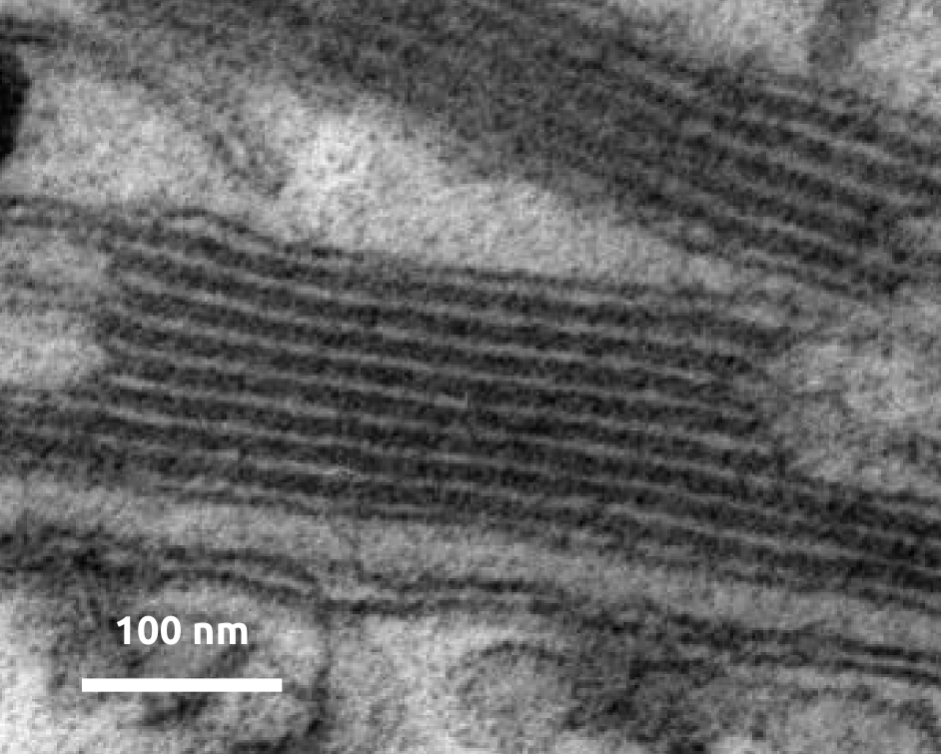
An example of nanostructured interfaces in an energy conversion device: thylakoids for photosynthesis (micrograph adapted and reproduced with author permission; (c) Andreas Anderluh and Bela Hausmann).

Despite the variety of physical states and chemical identities found in such energy conversion devices, they all share a fundamental principle: an increase of the geometric area of their interfaces should result in a commensurate increase in their throughput, until the concomitant increase in the diffusion distances of the charge carriers between the bulk and the interface causes transport to become limiting. For this reason, nanostructured interfaces with elongated folds or tubes can result in optimized devices (Figure 2).

In this context, any method capable of depositing thin functional layers onto structured substrates, and especially into nanoporous frameworks, is conferred with a direct relevance towards energy conversion applications. The conformal coating of non-planar samples is a property that uniquely defines atomic layer deposition (ALD) [3–7], which is why ALD is inherently suited to the preparation of energy conversion devices. ALD achieves a thin film growth by using well-defined surface chemistry. Two (or more) complementary, quantitative surface reactions performed subsequently and repeated in an alternating manner result in the deposition of a solid in a layer-by-layer fashion [8–10]. The surface chemistry is ‘self-limiting’: each reaction deposits an amount of material defined by the availability of surface reactive groups, not by the (local) partial pressure of gaseous precursors. This growth mode circumvents mass transport as the rate-limiting factor of the increase of the film thickness, thereby allowing for a homogeneous growth even if the gas phase is inhomogeneous – a situation notably found in highly porous systems.

Readers of this Thematic Series will obtain a glimpse of the broad applicability of the method in different types of energy conversion devices (summarized in Table 1). The plethora of functions which can be performed by ALD materials may be rationalized if a few common themes are recognized, which run like a common thread through this Thematic Series:

- ALD for a direct device function, such as light absorption in solar cells, ion conduction and electrocatalysis in fuel cells, or lithium uptake in batteries;
- ALD for separation and protection, in particular to prevent erosion or corrosion in electrochemical devices;
- ALD for interface engineering, for example defect passivation in solar cells or prevention of charge recombination by tunnel barriers, and for influencing the electronic structure of an underlying semiconductor.

**Table 1:** A non-exhaustive list of exemplary ALD applications in energy conversion devices illustrated in this Thematic Series and in previous literature. Reviews have been published recently on the applications of ALD in photovoltaics [11], lithium ion batteries [12], and solid oxide fuel cells [13].

Device type	Function of the ALD film	Literature references	References in this Thematic Series

Batteries	Li ion electrode materials	[14–16]	[17]
Batteries	Protective layer	[18–19]	
Batteries, fuel cells	Ion conduction	[20–22]	
Fuel cells, electrolysis, photoelectrolysis	Electrocatalysis	[23–26]	[27–28]
Photoelectrolysis, photovoltaics	Light absorber	[29–31]	
Photoelectrolysis, photovoltaics	Transparent conducting oxide	[32–33]	[34–35]
Photovoltaics	Electron conductor	[36–39]	[40–41]
Photovoltaics	Surface passivation	[42–44]	[45–46]
Photovoltaics	Tunnel barriers	[47–48]	

This Thematic Series will certainly provide the reader with novel ideas for exploiting ALD in the energy realm, and spur further original work in this rapidly developing research area. After its industrial application in electroluminescent displays, semiconductor logics (MOSFET), magnetic memory (TMR sensors) and semiconductor memory (DRAM), ALD has the potential to also become a critical tool in the area of energy conversion.

Julien Bachmann

Erlangen, November 2013
